# Light-Responsive Liquid Crystal Surface Topographies for Dynamic Stimulation of Cells

**DOI:** 10.1021/acsami.5c02526

**Published:** 2025-05-03

**Authors:** Ruth M.C. Verbroekken, Oksana K. Savchak, Thom F.J. Alofs, Albert P.H.J. Schenning, Burcu Gumuscu

**Affiliations:** 1 Stimuli-Responsive Functional Materials and Devices, Department of Chemical Engineering and Chemistry, Eindhoven University of Technology, P.O. Box 513, Eindhoven 5600 MB, The Netherlands; 2 Institute for Complex Molecular Systems, Eindhoven University of Technology, P.O. Box 513, Eindhoven 5600 MB, The Netherlands; 3 Biosensors and Devices Laboratory, Department of Biomedical Engineering, Eindhoven University of Technology, P.O. Box 513, Eindhoven 5600 MB, The Netherlands

**Keywords:** surface actuation, reconfigurable dynamic topographies, light-responsive liquid crystal polymers, mechanical cell-stimulation, materiobiology, fibroblast cells

## Abstract

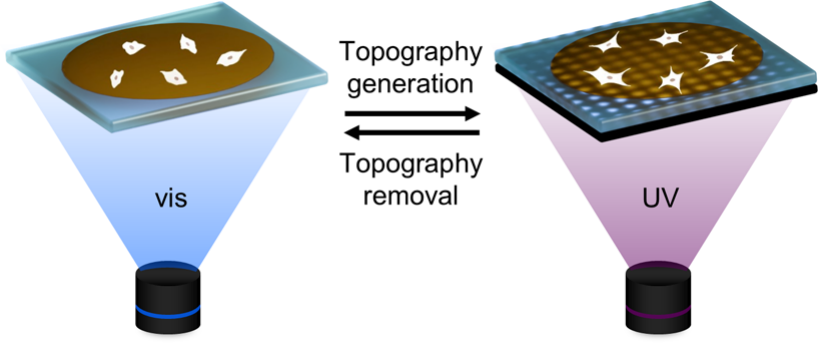

All biological surfaces possess distinct dynamic surface topographies. Due to their versatility, these topographies play a crucial role in modulating cell behavior and, when intentionally designed, can precisely guide cellular responses. So far, biomechanical responses have predominantly been studied on static surfaces, overlooking the dynamic environment in the body, where cells constantly interact with shifting biomechanical cues. In this work, we designed and fabricated a light-responsive liquid crystal polymer film to study the effect of micrometer-scale, dynamic surface topographies on cells under physiologically relevant conditions. The light-responsive liquid crystal polymers enable on-demand surface topographical changes, reaching pillar heights of 800 nm and grooved topographies with 700 nm height differences at 37 °C in water. The light-induced surface topographies increased mechanosensitive cell signaling by up to 2-fold higher yes-associated protein (YAP) translocation to the nucleus, as well as up to 3-fold more heterogeneity in distribution of focal adhesions, in a topography-related manner. The pillared topography was seen to cause a lower cellular response, while the grooved topography caused an increased mechanical activation, as well as cell alignment due to a more continuous and aligned physical cue that enhances cell organization. Excitingly, we observed that subsequent surface topography changes induced a 3-fold higher YAP nuclear translocation in fibroblast cells, as well as a 5-fold higher vinculin heterogeneity distribution, indicating that multiple cycles of topography exposure ampliated the cell response. Our work emphasizes the potential of light-responsive liquid crystal polymer films generating dynamic biomechanical cues that allow us to modulate and steer cells in vitro.

## Introduction

All tissues exhibit unique and dynamic topographies that are continually remodelled based on their function and location. The remodeling of the topographies is mostly regulated by the cell-extracellular matrix (ECM) interactions at the nanoscale. Both ECM and cells influence the behavior of each other to adapt to (patho)physiological occurrences, such as disease, regeneration, and healing. Mechanical stimuli have an important role in such occurrences as they are majorly responsible for the modulation of the cell behavior and phenotype. For example, in tissue regeneration, topographical features can influence cell behavior by promoting a healing response, but they can also trigger increased inflammation and lead to scar formation.^1−5^ Fibroblasts, major players in the tissue regeneration processes, have been reported to be highly affected by mechanical cues of a topography-covered surface.^4,6−11^ Surface topographies have been shown to promote specific phenotypes in fibroblasts that fundamentally affect their gene expression, cell cycle, reactivity, and cell morphology.^4,12,13^ Understanding how surface topographies influence cell responses might lead to accurate cell behavior modeling and improved outcomes for handling (patho)physiological occurrences. Despite the dynamic nature of the tissue regeneration and remodelling, current research has predominantly focused on static topographies,^12−16^ which fail to fully reflect the dynamic nature of the ECM. Hence, on-demand, dynamic surface topographies are required to mimic and stimulate physiologically relevant cellular responses.

Light is an attractive stimulus for generating dynamic surface topographies, offering unmatched precision and spatiotemporal control.^17−24^ Up to now, light-responsive hydrogels^18,19,25−27^ and liquid crystals (LCs)^20,21,23^ have been used to create dynamic surface topographies. While responsive topographies in hydrogels are created based on the principle of swelling and deswelling driven by water transport,^26,28^ LC polymers undergo stimuli-induced shape changes based on their change in molecular order. So far, light-responsive hydrogels for cell manipulation have been rarely reported. The dynamic induction of surface topographies can condition cell responses and initiate mechanotransduction pathway events.^29−31^ LC polymer-based static surface topographies have already served as excellent platforms to guide cell migration based on cell-material interfaces, aligning cells,^32,33^ inducing strain,^34,35^ and affecting cell migration.^22^ Although irreversible-dynamic topographical LC surface features show promising results on material-induced cell behavior, fully reversible and reconfigurable topographies of light-responsive liquid crystal surfaces for dynamic stimulation of cells have not been reported yet.

**Figure 1:**
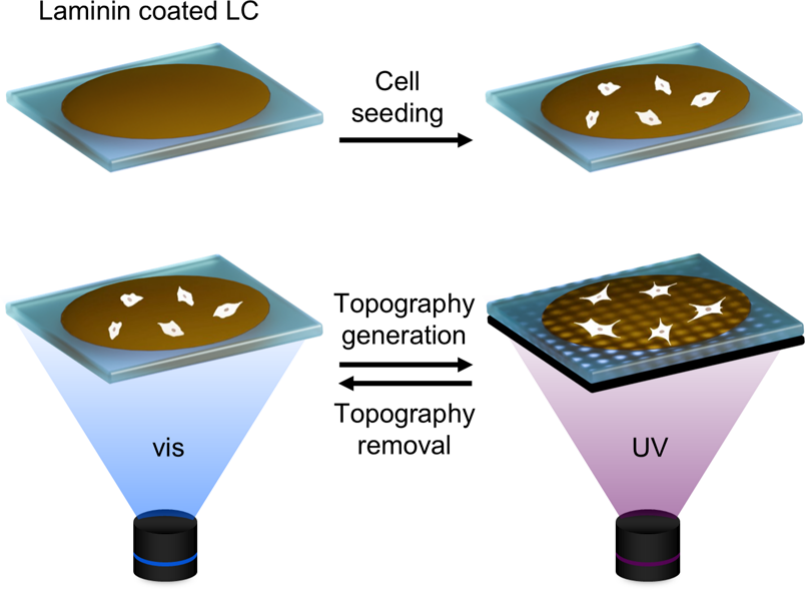
Scheme showing the working principle of LC film functionalization and creation of dynamic topographies by using a photomask. Laminin coated LC films allow for cell (fibroblast) attachment and growth. 24 h post seeding, bottom mask-exposed UV illumination yields surface topographical features, that, upon full visible illumination, is removed. Not to scale.

In this work, we report a biocompatible, light-responsive LC film that enables on-demand, reconfigurable topographical features in a physiologically relevant cell microenvironment. A key advancement in our work is that light actuation allows biomechanical responses to be observed at constant physiological temperatures, avoiding the interface heating associated with other stimuli. We created dynamic surface topographies (Figure 1) by fabricating light-responsive LC films containing azobenzene photo-switchable molecules.^21−24,36^ The LC surface has been optimized for demonstrating the largest actuation at 37 °C in water and subsequently functionalizing it with a cell-native surface protein (i.e., laminin). The resulting material allows for on-demand actuation of micrometer-scale surface topographies using photopatterning. With high design flexibility guided by the photomask, the light-responsive LC films provide a biocompatible platform to study dynamic cell-material interactions in the context of mechanotransduction *in vitro*. Pillared and grooved topographies were selected as they are expected to evoke different cellular responses.^18,37−44^ The dermal fibroblast response was steered in three manners by dynamic surface topographies with maxima of 800 nm peak-to-valley distances. The fibroblasts on LC films adapt their cell morphology, alignment, and attachment pattern depending on the induced reconfigurable surface topographies. Therefore, light-responsive LC films offer an attractive approach to study the time-dependent effect of dynamic topographies on cellular processes.

## Results and Discussion

### Fabrication of Light-Responsive Liquid Crystal Films

**Figure 2:**
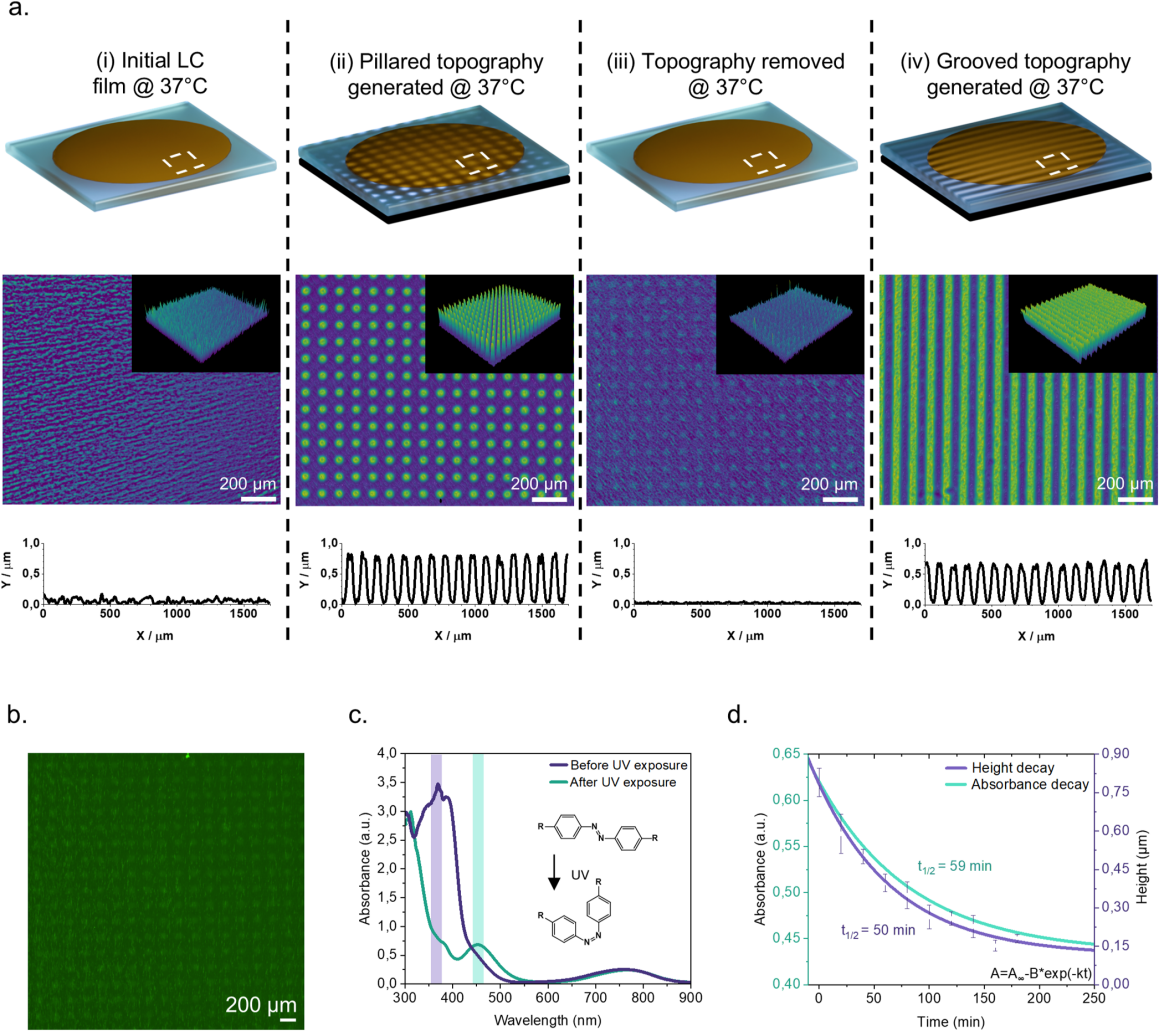
(a) Scheme illustrating an LC film in air at 37 °C, starting with a flat surface (i) and next with a pillared surface topography (ii) (800 nm peak-to-valley height, 50 μm diameter features, and 50 μm spacing). Sequential visible light exposure resets the pillared features to a flat surface (iii), after which a second mask induces a grooved surface topography (iv) (700 nm peak-to-valley height, 50 and 50 μm spacing). (b) Fluorescence microscopy image of a pillared topography LC film displaying fluorescence at the exposed regions. (c) Absorbance spectrum of the light-responsive LC film before (purple) and after (green) UV light exposure. The absorbance spectrum after UV exposure shows diminished absorbance at 365 nm and increased absorbance at 455 nm. (d) Plot of the cis-azobenzene decay (green), together with a plot of the corresponding topographical height decay over time (purple) of a LC film in the dark, in air at 37 °C. Both fitted with an exponential decay function *A* = *A_∞_–Be*^*–kt*^, resulting in a half-life time of 59 and 50 min, respectively.

The light-responsive LC film was prepared from a mix of liquid crystalline mono- and diacrylate monomers (**1,2,3**), an acrylate functionalized photoisomerizable azobenzene (**4**), a chiral dopant for creating a cholesteric LC phase (**5**), chain transfer agent (**6**), and a photo initiator (**7**) (SI, Figure S1).^20,23,24,45^ The addition of the chiral dopant ensures the predominant actuation of the film in the direction of the helical axis. Due to the out-of-plane directionality of the helical structure of the LC, the film predominantly expands in the direction perpendicular to the plane when illuminated. The use of a high ratio mono- to diacrylate LC monomers^20,23,24^ and the incorporation of a dithiol that acts as a chain transfer agent,^45^ lowers the LC transition temperatures and ensures reversible actuation at physiological conditions (*vide infra*). In-plane shearing of the LC mix in the cholesteric phase between an acrylate functionalized and a fluorofunctionalized glass plate allowed for the required planar cholesteric LC alignment. Sequential photopolymerization (455 nm light) at 30 °C, followed by a post-baking step at 80 °C, yielded a fully polymerized planar cholesteric light-responsive LC film (Figure 2a(i)) of 20 μm as dictated by the diameter of the spacer beads. The disappearance of the peaks for the free acrylate groups at 1410 and 810 cm^–1^ in the Fourier-transform infrared spectroscopy (FT-IR) spectrum (Figure S2a) confirms full polymerization.^46^ The obtained LC film allowed for actuation at physiologically relevant temperatures, showing a glass transition temperature below 37 °C and a nematic-to-isotropic transition temperature of around 90 °C (Figure S2b,c). The resulting LC film is functionalized with laminin to promote cell-adhesion and approach a more natural cell environment.

### Light-Responsive Reconfigurable Surface Topographies

Mask illumination from below of the laminin functionalized LC film at 37 °C in an aqueous environment (i.e., physiological condition) allowed for temporary topographies on a micrometer scale with high design flexibility. Selective illumination through a rectangular patterned photomask with 50 μm diameter holes with 50 μm interspacing placed against the glass substrate (Figure 1) generated maximized surface topographies in a perpendicular direction to the plane. Optimized mask-exposed UV (365 nm) illumination for four min at a low light intensity (10 mW/cm^2^) resulted in surface topographical deformations of 4%, achieving mask corresponding topographies with peak-to-valley heights of up to 800 nm (Figure S2d). Such 800 nm peak-to-valley heights are known to be sufficient acting as mechanical surface stimuli to exposed cells.^25,47,48^ Interestingly, the generated topographies in air and at 37 °C are identical to those at physiological conditions, which indicates there is no photothermal contribution to topography generation (Figure 2a(ii) versus Figure S 2d).^49^ The azobenzene photoisomerization was accompanied by a change in LC film stiffness as measured on the laminin functionalized LC film, decreasing from 1.8 to 1.2 GPa upon actuation as measured by atomic force microscopy (AFM). The stiffness modulus change is not expected to cause any pronounced effect on the cell response due to the overall stiff surface of the LC film (Supporting Information). The photoisomerization did not affect surface wettability, as evidenced by a consistent water contact angle of 66° before and after actuation (Figure S2e). Fluorescence microscopy revealed a weak fluorescent signal in the exposed surface regions, likely caused by the fluorescent *cis*-azobenzene state^50^ (Figure 2b). The fluorescent signal depicting generated surface features allows for the precise locating of cells, allowing us to accurately track cell behavior with respect to generated topographies.

For convenience, we studied the regeneration of topographies in air at 37 °C. To remove the surface topographies on demand, the LC film was illuminated from the bottom with ten min visible light (455 nm) at a low intensity (10 mW/cm^2^). This removal confirmed the reversibility of the surface topographies dictated by switching of the azobenzene isomeric state (*vide infra*). The visible light exposure triggered the topography reconversion with a nearly full conversion rate, as confirmed with white-light interferometry and UV absorbance data (Figure 2a(iii),Figure S2f). After the topography was removed, a new cycle of bottom mask UV illumination yielded new surface topographies, such as the 700 nm peak-to-valley grooves presented in the second topography generation cycle using a mask with groove topographies with 50 μm lines with 50 μm interspacing (Figure 2a(iv)).

### Light-Switching Mechanism

UV exposure to the LC film triggers a *trans-*to*-cis* photoisomerization of the azobenzene molecule (Figure 2c), resulting in a change of the molecular order of the exposed region.^21−24,36^ Photoexcitation causes *trans-*to*-cis* isomerization, where the azobenzene isomeric change can be observed by tracking the azo-specific absorbance via UV–vis spectroscopy. Whereas the *cis*-azobenzene displayed a stronger absorbance in the visible range (455 nm), the *trans*-azobenzene absorbs light in the UV regime, showing a strong absorbance at a wavelength of 365 nm (Figure 2c). Evident from the absorbance spectrum of an LC film, UV exposure yielded *cis*-azobenzene-specific absorbance at 455 nm. Sequential visible light exposure triggered back-conversion to the thermodynamically more stable 365 nm absorbing *trans*-azobenzene, flattening the LC film.

It is known that *cis*-azobenzene isomerizes back over time to the thermodynamically more favorable *trans*-isomer.^50,51^ Azobenzene back-isomerization was observed with the overtime *cis*-related absorbance band decay at 455 nm (Figure S2g), with an LC film in air at 37 °C in the dark. Therefore, recording the absorbance at 455 nm is indicative of the *cis*-azobenzene population inside the film. When measured over a time span of 15 h, this *cis*-specific absorbance band showed a strong decay within the first two 3 h (Figure 2d), indicating the *cis*-to-*trans* back-isomerization. The *cis* decay was quantified by fitting the data to a single-exponential decay function (*A* = *A*_∞_ – *Be*^–*kt*^),^52^ determining a half-life time of 59 min. To relate the *cis* decay to the decay of surface topographies, in addition, the topographical decay was measured over time. For this measurement, a pillared surface was generated, after which the decay of the topographies in air at 37 °C was measured over time. When measured over a time span of 2.5 h, the topography height showed a strong decay following the decay trend of *cis-*azobenzene (Figure 2d). Fitting the height decay to the same single-exponential decay function (*A* = *A*_∞_ – *Be*^–*kt*^),^52^ determines a topography half-life time of 50 min, closely corresponding to that of the decay of *cis*-azobenzene. This correspondence revealed that the topographical changes are related to the *cis*-azobenzene content, suggesting a photochemical actuation mechanism.^49^ We established the cell exposure time to actuated LC film surfaces prior to imaging at 3 h per exposure condition, due to the peak-to-valley height difference of 180 nm three h post surface topography generation. Three hours of cell exposure to the activated LC film surface resulted in a sufficient time for the cells to adapt to surface topographies.^25,48^

### Cell Viability, Morphology, and Mechanosensing on LC Films

**Figure 3:**
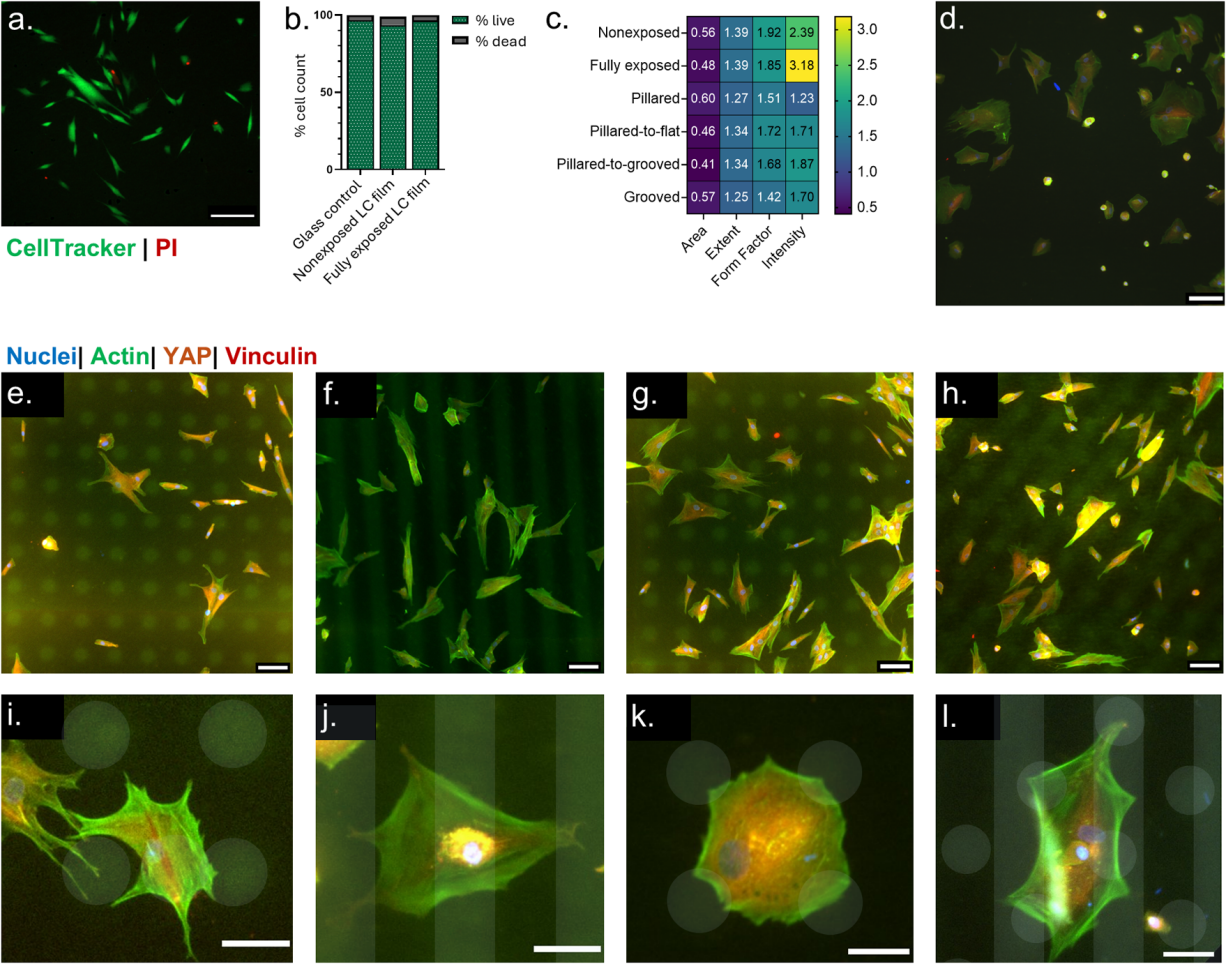
Fibroblast response to LC film actuation and dynamic topography formation. (a) Representative image of live/dead staining on a fully light exposed LC film. Live cells are stained green and dead cells are stained red. Scale bar is 200 μm. (b) Cell viability quantification on glass control surface, nonexposed LC film, and fully exposed LC film. Green regions represent the percentage of the live population, gray region represent the percentage of dead cell population. (c) Heatmap of fibroblast morphological features. All conditions are normalized to the glass control. (d–h) Human dermal fibroblasts. Nuclei stained with blue, actin cytoskeleton in green, YAP protein in orange, and vinculin in red. All scale bars are 100 μm. (d) Fibroblasts on nonexposed LC film surface, (e) fibroblasts on the pillared topography, (f) fibroblasts on the grooved topography, (g) fibroblasts on the pillared-to-flat topography, and (h) fibroblasts on the pillared-to-grooved topography. The observed grooves’ rotation is due to the sample positioning during exposure and imaging (i–l) Close up of human dermal fibroblast interaction with the surface topographies. All scale bars are 50 μm. Semitransparent geometric shapes represent the real location of the surface topography on the LC film. (i) Fibroblast cell interaction with pillared topography. (j) Fibroblast cell interaction with grooved topography. (k) Fibroblast cell interaction with pillared-to-flat topography. (l) Fibroblast cell interaction with pillared-to-grooved topography.

We first characterized the cell response to flat nonexposed and fully exposed LC films in order to understand the contribution of the LC film and UV light exposure to the cell response. The cell viability on the LC films was assessed by live/dead staining and compared to that on traditionally used coverslip glass surfaces. Figure 3a shows the results of the live–dead assay, with living cells stained green and dead cells stained red. After 30 h in culture, comprising 24 h for cell resting and attachment plus the time required for completing two actuation cycles, cell viability on the nonexposed glass coverslip surface was 96.6%. In comparison, cells on the nonexposed LC film showed 93.4% viability, while those on the double fully UV-exposed LC film had a 96.4% viable population (Figure 3b). The comparable number of viable cells present in each condition demonstrates the biocompatibility of the LC film and confirms the hypothesized biocompatibility of the UV light-based actuation. This biocompatible UV actuation is supported by the light attenuation in the 20 μm-thick LC film, minimizing the exposure to light at the cell-LC surface.

In order to understand and quantify cell response to LC film material and dynamic topography generation, we assessed three main parameters: (1) change of the cytoskeleton morphology, (2) upregulation of the mechanosensitive yes-associated protein (YAP) and the transcriptional coactivator with PDZ-binding motif (YAP/TAZ) complex, and (3) expression and redistribution of focal adhesions. Cell morphology was assessed with four main morphological cell features to explore the fibroblast response (Figure 3c). First, we evaluated the cell area, which describes the size of the cell body size. The area of fibroblasts grown on the LC film was notably lower than that grown on the glass surface. The nonexposed control showed a 0.57 ratio-fold reduction in the cell area (SD = 0.26), demonstrating the fibroblasts’ tendency to reduce in size when grown on LC films as compared to the glass surface (AVG = 0.94, SD = 0.38). The fibroblasts on a glass surface (Figure S3a) had a large (100–200 μm in diameter) and spread-out cell body that followed a more elongated shape. In contrast, cells on nonactivated LC surfaces (Figure 3d) exhibited a distinct morphology and size, with the majority of the population significantly smaller, displaying cell body diameters of 20–30 μm. Meanwhile, the remaining cells, despite having larger areas (∼100 μm), retained a more circular morphology compared with the glass control. The fully exposed LC control also shows a reduction in the cell area with an average ratio of 0.48 (SD = 0.20). Both the nonexposed and fully exposed LC films showed a more regular cell shape with a more uniform surface containing fewer protrusions and irregularities (AVG = 1.39, SD = 0.13; AVG = 1.39, SD = 0.11; respectively) as assessed by the extent parameter. Nonexposed (AVG = 1.92, SD = 0.54) and fully exposed LC films (AVG = 1.85, SD = 0.42) also presented a more circular shape, assessed by the form factor. All morphological parameters were normalized to the glass control as the fibroblast morphology reference and presented in Figure S3b. Overall, nonexposed and fully exposed conditions show no significant difference in the morphology parameters, with a high standard deviation and closely related cellular change at all morphological aspects. The morphological similarity indicates that the fibroblast morphology is mainly defined by a common LC film surface characteristic independent of the actuation state. When further looking into the cell cytoskeleton, actin intensity exhibits the most notable difference between the controls. The nonexposed control shows a 2-fold increase (AVG = 2.4, SD = 0.94), while the fully exposed control shows a 3-fold increase (AVG = 3.18, SD = 1.56) in the actin intensity. Altogether, higher actin intensity can be due to the smaller cell size that causes a higher degree of compactness of the actin bundles as well as reflects the cytoskeletal organization and dynamics within the fibroblasts.^53^

**Figure 4:**
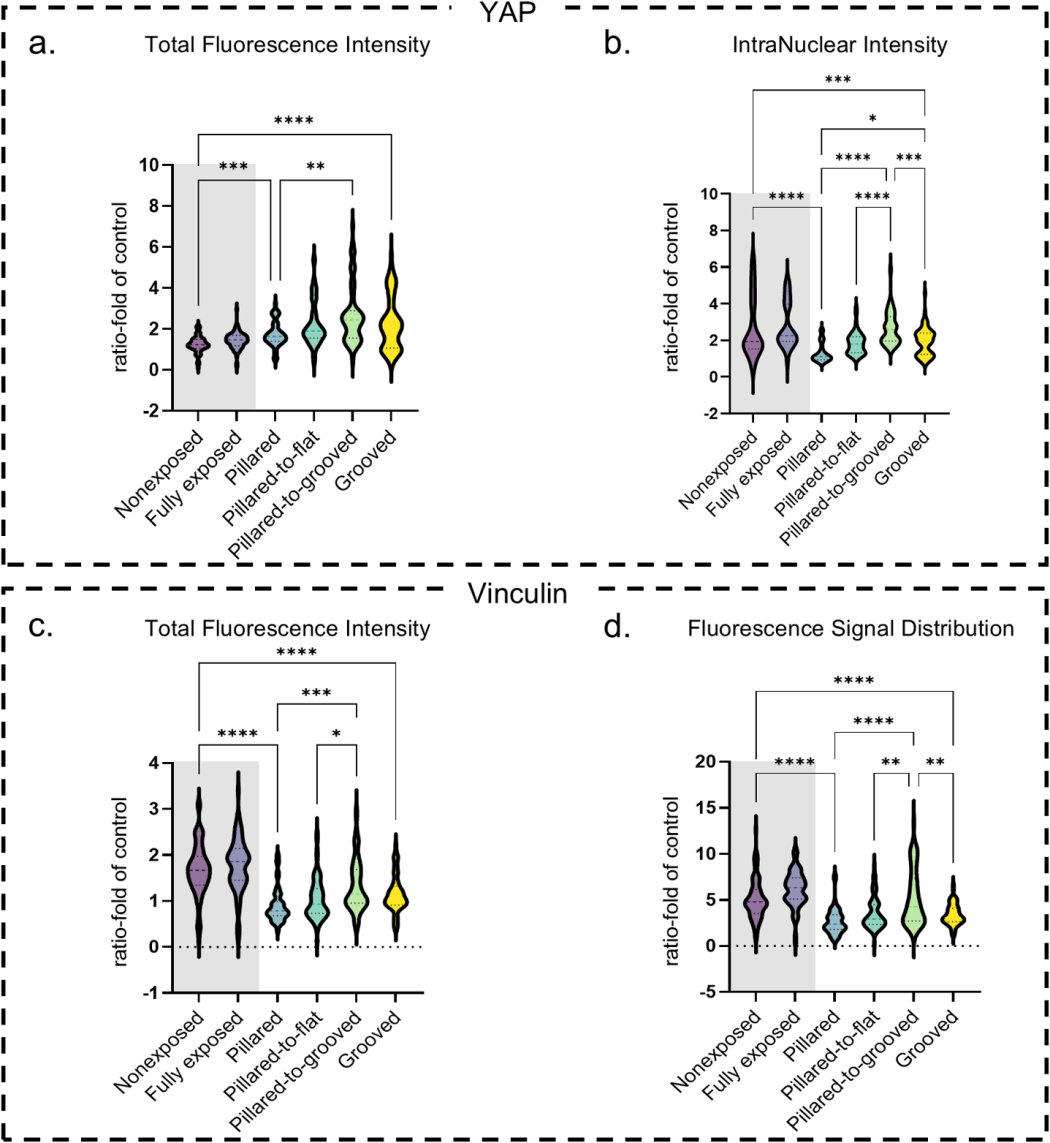
Fibroblast mechanosensitive response and focal adhesions. Gray shading highlights the control conditions. All conditions normalized to glass control and represented in a violin plot with the mean and median values, as well as data distribution. *N* = 3, * *p* < 0.05, ** *p* < 0.01, *** *p* < 0.001, **** *p* < 0.0001. (a) Total fluorescence intensity of YAP staining. (b) Nuclear intensity of the YAP staining. (c) Total fluorescence intensity of vinculin staining. (d) Median absolute deviation of the intensity of vinculin staining.

We assessed the fibroblast response to the LC film surface in terms of mechanosensitivity by quantifying YAP complex expression and its translocation to the nucleus. YAP nuclear translocation is typically used as an assessment of the cell mechanical sensing activation.^54^ Activated by mechanical stress, YAP translocates to the nucleus where it influences gene expression related to cytoskeletal dynamics and integrin signaling. This regulation is essential for altering cell morphology and enhancing focal adhesion formation, thereby affecting cell shape, migration, and adhesion strength.^55,56^ The quantification of the total YAP protein intensity in the fibroblast cells (Figure 4a) showed that both the nonexposed control (AVG = 1.25, SD = 0.4) and fully exposed control (AVG = 1.44, SD = 0.41) result in higher YAP intensities when compared to the glass control condition used as a reference (Figure S3c). However, there is no significant difference between the nonexposed and fully exposed controls, as well as the average intensity is lower than that of the topography conditions. Interestingly, both conditions show a high degree of YAP nuclear translocation with an average of 2.7-fold (SD = 1.6) increase at the no-exposure condition and 2.8-fold (SD = 1.2) increase at the full-exposure condition (Figure 4b). Likewise, the total vinculin fluorescence intensity (Figure 4c) shows a similar pattern of behavior to that of the nuclear YAP intensity. When seeded on LC films, cells express more vinculin when compared to the glass control (AVG = 1.00, SD = 0.31, Figure S3c) with the nonexposed control condition denoting a 1.7-fold increase (SD = 0.56) and the fully exposed control showing a 1.8-fold increase (SD = 0.58) in vinculin protein expression. Furthermore, both the nonexposed condition (AVG = 5.09, SD = 2.17) and fully exposed condition (AVG = 6.17, SD = 1.94) showed an increase in the vinculin distribution heterogeneity when compared to the glass surface (AVG = 0.97, SD = 0.23) (Figure 4d, Figure S3c). This distribution heterogeneity suggests that fibroblasts have preferential adhesion points even on the flat surfaces of the LC films. Vinculin is a major regulator of focal adhesion formation. When cells do not have any binding preference, as an example of a smooth glass surface, focal adhesions are evenly distributed over the cell-surface interface. However, in case the cells experience any differential stimulus from the surface, they can react by preferentially binding to the different regions of the surface. This preferential binding creates a higher distribution, as more focal adhesions will be concentrated at the interfaces where the cells are more directed or prone to bind.^57,58^ Overall, the cells on flat LC films, both fully exposed and nonexposed, appear to have a high degree of mechanical stimulation and focal adhesion reorganization. However, while both conditions are significantly different when compared to the glass control, we consistently observed no significant difference in morphology, mechanical activation, or adhesion between the nonexposed and fully exposed conditions. Altogether, the results indicate that the observed response is consistent across flat LC films regardless of the actuation state.

### Dynamic Generation of Surface Topographies

We evaluated the cell response to surface topographical changes using the two distinct topographies: pillared and grooved. As mentioned in the introduction, pillar and groove topographies offer the benefit of distinct cellular responses widely reported in the literature, making them suitable for exploring cell responses to novel materials. Furthermore, a 50 × 50 μm topography spacing was selected to match the spacing of the topography to the average size of the cell to guarantee that each cell will be mechanically stimulated and able to detect topographical features in its vicinity. The effect of a single topography induction on the cells was assessed 3 h after topography formation, by which time the LC topography’s half-lifetime would have caused it to decay from 800 nm to approximately 180 nm in height. The cell body area under the pillared condition had an average of 0.60-fold decrease (SD = 0.34), while the grooved condition had an average 0.57-fold area decrease (SD = 0.32). The cells furthermore showed regular and smooth morphologies (AVG = 1.27, SD = 0.15 for pillared; AVG = 1.25, SD = 0.09 for grooved) with more circular shapes (AVG = 1.51, SD = 0.36 for pillared; AVG = 1.42, SD = 0.29 for grooved). Morphology-wise, there was no distinct difference in cell response to different topographies. However, the actin intensity showed a different trend: While a pillared topography shows a minor increase (AVG = 1.23, SD = 0.30), the grooved topography shows a significant increase (AVG = 1.70, SD = 0.96). Such an increased actin intensity could be indicative of a higher cell response to the grooved topography, possibly leading to a higher degree of cell restructuring and alignment to the grooves. Cell morphology can also be assessed visually in relation to the generated topographies (Figures 3e–h). The fluorescence of the topography-covered LC film regions, dictated by the patterning photomask, enables the determination of individual cell responses to the given topographies. On pillared topography-covered LC surfaces (Figure 3e), cells preferred to stay in the exposed areas of the film, attaching themselves to the pillars and spreading out across the surface. Furthermore, in a close-up image (Figure 3i) it can be seen that a cell is interacting with the surface pillar topography, protruding in a way to “wrap” around the pillar. This cell behavior suggests that the cells have a higher binding affinity for the exposed areas, resulting in strong interactions with the pillar structures. This also causes the observed formation of cell protrusions in the direction of the pillared topography. On the other hand, when exposing the cells to a grooved topography (Figure 3f), a portion of the cell population appears to align itself with the grooved topography. This alignment suggests that the cells respond to the directional cues of the topography, guiding their orientation and elongation along the grooves. Moreover, when looking at the cell protrusion pattern, it can be seen that while extending over multiple grooves, the cells bind preferentially to the exposed area of the LC film, the top of the groove (Figure 3j).

When assessing the mechanosensitive signaling of the fibroblasts on topographies, cells on topography-covered LC films showed a distinct change, demonstrating an increase of 1.73-fold (SD = 0.61) of the YAP intensity for the pillared topography and 2.27-fold (SD = 1.48) for the grooved topography (Figure 4a). This increase suggests that LC film topographies induce an increase in the level of YAP protein expression. Differences in cell behavior related to the topography were more clearly observed in the nuclear translocation of the YAP signal (Figure 4b). Cells on a single topography exhibited a distinct response in the nuclear translocation of YAP. As such, the pillared topography resulted in a slight, but not significant, increase in the nuclear translocation of the cells. The grooved condition, on the other hand, resulted in a significantly higher increase in YAP nuclear translocation (AVG = 1.93, SD = 0.75) compared to both the glass and pillared conditions. This demonstrates the cells’ ability to respond differentially to various topographies and highlights fibroblasts’ stronger mechanical sensing and interaction with the grooved topography. Additionally, we studied how fibroblasts remodel their attachment in response to a dynamically changing environment. The pillared topography showed a slight decrease in total vinculin expression (AVG = 0.89, SD = 0.37), whereas the grooved topography (AVG = 1.15, SD = 0.39) showed a significant increase (Figure 4c). Both topographies also demonstrated a higher vinculin intensity distribution, indicating that the fibroblasts have preferred attachment points over the LC surfaces. The pillared topography (AVG = 2.69, SD = 1.39) and grooved topography (AVG = 3.39, SD = 1.13) both showed a significant increase in vinculin distribution but no difference between the topography conditions themselves (Figure 4d). The vinculin expression and distribution are known to be influenced by topographical features in fibroblasts.^12,18^ These vinculin results show that even though the cells bind more heterogeneously to the topography-covered surface, there are no detectable differences in the focal adhesion distribution. We hypothesize that due to the consistent spacing between the pillars and grooves (50 μm), cells always have a comparable area of activated surface nearby for binding. Overall, the different topographies induce distinct responses in fibroblast mechanical signaling, actin polymerization, and remodeling. While no notable differences are observed in morphological and focal adhesion patterns, we attribute this to the spatial similarity of the topographies, as both the pillared and grooved topographies share the same size and interspacing.

### Double-Topography Exposure and Cell Memory

To explore the fibroblasts’ ability to adapt to new topographical cues and assess cell memory, we examined their response over prolonged exposure to different topographies. Cells have mechanosensitive receptors, mainly integrins and ionic channels. Activation of these mechanosensitive receptors leads to a cascade of signaling events as well as alterations in genetic expression and protein post-translational modifications.^59,60^ Therefore, even a short mechanical stimulus can fundamentally alter the cell response and morphology in the long term.^61^ Initially, we induced the pillared topography, allowing the cells to adapt to this surface change for 3 h. After this period, we reset the surface and directly induced the grooved topography. To assess the cell memory effect, after three sequential hours of adaptation on the grooved topography, we evaluated whether the cells returned to their original morphology, as observed in the nonexposed LC control. When the cell area was examined, both conditions showed a decreased average cell area. The extent of both double exposure conditions showed the same behavior pattern of more regularly shaped cells without any protrusions when compared to the glass control, yet no significant difference from any of the other LC film conditions. The form factor showed a significant increase in cell circularity, with the pillared-to-grooved (AVG = 1.68, SD = 0.23) and pillared-to-flat (AVG = 1.71, SD = 0.26) conditions, resulting in a more rounded morphology (Figure 3c). Interestingly, while LC films promote more circular morphologies, a comparison of the different topographies reveals that the grooved topography induces less circularity than the pillared topography. This may be due to the grooves facilitating fibroblast alignment along their structure. Furthermore, the intensity parameters reflect the previously discussed trend of the highest degree of variability as well as following the same pattern as the mechanical activation. Consequently, the pillared-to-flat condition showed only a negligible increase in the actin intensity (AVG = 1.71, SD = 0.44) when compared to the single-grooved condition, while an induction of a secondary topography in the pillared-to-grooved condition (AVG = 1.87, SD = 0.52) caused a notable increase in the actin intensity. Cell morphology and memory are further illustrated in Figure 3g, where fibroblasts exposed to the flat surface for the same duration as the pillared topography still retained the morphology observed on the single-pillared topography. Nevertheless, as demonstrated in Figure 3k, although the cells maintain the morphology and attachment pattern acquired during exposure to the pillared topography, this response becomes less evident as the cells adjust to the flat surface. In the double-topography condition (Figure 3h), the grooved topography seemed to override the pillared topography, with most of the cell population aligning along the grooved topography. Moreover, more cells appear aligned to the topography when compared to the grooved topography (Figure 3h), suggesting that the cells may respond more strongly to secondary stimuli.^18^ Notably, while aligning to the grooved topography, the cells preserve the cell body protrusions affiliated with the pillared topography, indicating the presence of a cell memory effect (Figure 3l).

When the mechanosensitive response of the fibroblasts was looked at, the total YAP intensity did not increase without additional topographical actuation. The pillared-to-flat condition showed a 2.21-fold increase in intensity (SD = 1.01), a value not significantly different from that of any of the single topography conditions. However, when the cells were exposed to a second cycle of topographies, as in the pillared-to-grooved condition, we observed the highest increase in YAP intensity (AVG = 2.68, SD = 1.43), which is significantly higher than the pillared topography (Figure 4a). The increased YAP intensity suggests that double exposure to topographical features, rather than time, further enhances YAP expression in fibroblasts. Furthermore, in the YAP nuclear translocation, the pillar-to-flat condition was comparable to the pillared one, showing a nonsignificant increase (AVG = 1.85, SD = 0.62). On the other hand, the pillared-to-grooved condition showed the highest nuclear translocation value (AVG = 2.67, SD = 0.93), consistent with the expected result of double-topography induction activating more mechanosensitive signaling pathways in the cell (Figure 4b). When assessing the vinculin expression, the pillared-to-flat control (AVG = 1.04, SD = 0.45) showed no significant difference in vinculin intensity compared to the pillared topography. However, the pillared-to-grooved condition showed the greatest increase among the topographically actuated LC films, with an average value of 1.33-fold increase. The vinculin intensity in the pillared-to-grooved condition additionally shows an increase, although not statistically different, when compared to the grooved topography (Figure 4c). This suggests that focal adhesion formation is induced by the topographical features and not solely by a more prolonged cell seeding period. Furthermore, the vinculin intensity distribution (Figure 4d) shows that the pillared-to-flat condition (AVG = 3.53, SD = 1.70) has no significant increase or decrease in the focal adhesion distribution, meaning that fibroblasts retain their original focal adhesion pattern from the first topography exposure. However, when the fibroblasts were exposed to a second topography in the pillared-to-grooved condition, we observed a 5.22-fold increase in vinculin heterogeneity (SD = 2.98). This is consistent with the total vinculin intensity result, as the cells are forced to adapt to the new topography by increasing the formation of focal adhesions and altering their distribution patterns. Altogether, these findings in YAP nuclear translocation, focal adhesion expression, and distribution are in line with previously reported studies.^12,58,62^

## Conclusions

We designed a light-responsive LC film that allows for precise topography generation and functions effectively in a physiological environment. The reversible nature of the generated LC topographies mimics the dynamic ECM surrounding. Therefore, the laminin functionalized light-responsive LC films overcome the lack of dynamic function of the static cell culturing platforms often resourced when studying mechanically stimulated cell behavior. With surface topographies as high as 800 nm, the LC film demonstrates a new approach toward precise understanding and modeling of cellular behavior with respect to dynamic mechanical surface changes. The new approach toward engineering regenerative topographies allows for dynamic mechanical stimuli in a biocompatible platform. Bottom illumination of the setup ensures light absorbance by the azobenzene-containing LC film, therefore blocking the light from the cell side and hence largely protecting the cells from irradiation. Furthermore, the LC film has a high design flexibility, topographical patterning that is visible under fluorescence microscopy, allowing the exact pinpointing of the cell with respect to the generated surface topographies.

*In vitro* studies demonstrate that LC actuation is cell-compatible, with no decrease in cell viability on LC film surfaces even under UV illumination. Furthermore, cells exhibit distinct morphological changes and topography-driven alterations in mechanosensitive YAP/TAZ protein nuclear translocation and focal adhesion patterns. Notably, fibroblasts respond more strongly to grooved topographies than to pillared ones, showing upregulated mechanosensitive signaling and increased vinculin expression. Specifically, grooved topographies induce cell alignment, while pillared topographies cause cell protrusions toward the pillars. Additionally, double exposure to topographies enhances cell response, significantly increasing YAP expression, nuclear translocation, and vinculin distribution. This indicates that cells align to the second topography, demonstrating fibroblast plasticity in adapting to new topographies. Moreover, fibroblasts exhibit a memory effect, retaining initial binding points to pillars even on flat surfaces. Finally, the high mechanical response of flat LC surfaces suggests potential for future studies on the detection capabilities of biological cells under minimal mechanical stimuli. The system offers a platform that mimics the dynamic extracellular matrix (ECM) with high design flexibility, dynamic surface features allowing for the study of varying cell types and cellular processes.

## Experimental Section

### Preparation and Characterization of LC Film

See the SI.

### LC Film Functionalization

To accommodate cell culturing, LC films were encompassed by a PDMS well attached to the glass surface. LC films were sterilized by 15 min incubation with 70% ethanol, followed by 15 min evaporation and 4 consecutive washes of the surface with sterile Milli-Q water. The surfaces were then coated with a 5 μg/mL laminin solution in Milli-Q water for 1 h at room temperature and protected from the light. After laminin coating, LC films were carefully washed 1× with sterile PBS solution and immediately used for cell seeding. The coating protocol was identical for the coverslip glass experiment, which served as a control group.

### Actuation of the LC Film

Reconfigurable surface topographical patterning was achieved by UV mask illumination of the LC film. The patterning masks, chrome masks, are obtained from the JD photo data. Sequential topography removal took place via full visible illumination. For the illumination, 365 nm (4 min, 10 mW/cm^–2^) and 455 nm (10 min, 10 mW/cm^–2^) light-emitting diode (LED) lamps were used (obtained from Thorlabs M365L2 and M455L3, respectively), both mounted with a Thorlabs COP1-A collimator. Induced surface topographies were measured via white-light interferometry using the Sensofar S Neox white-light&nbsp;interferometer.

### HDF Cell Culture and Cell Seeding on the LC Film

Human Dermal Fibroblasts were purchased from Lonza. Cells were cultured in DMEM media (D5796, Sigma-Aldrich) supplemented with 10% fetal bovine serum (Serana, origin Brazil) and 1% Penicillin/Streptomycin (Gibco) and passaged until the maximum passage number was 12. Cells were seeded on the LC film surface at a density of 5000 cells/cm^2^, supplemented with 10 ng/mL TGF-β and left to attach and differentiate for 24 h prior to the film actuation. Subsequently, the LC films were actuated correspondingly to the condition, and the cells were transferred to the cell incubator for 3 h in the dark. Single exposure conditions were immediately fixed after 3 h, while control conditions and double exposure conditions were reset back to flat film and re-exposed correspondingly to the condition. After three more hours, these conditions were also fixed.

### Cell Viability Staining

Cell viability was quantified by performing a live–dead staining assay. For that, after 30 h in culture, glass control, no-exposure LC film, and full-exposure LC film condition samples were washed with sterile PBS and incubated with 10 μM CellTracker Green (Invitrogen) in PBS for 30 min in the incubator. After, the cells were washed again with sterile PBS and incubated with 10 μM propidium iodide (PI, Invitrogen) in PBS for 10 min and imaged immediately. For each condition, 20 images at 10× objective were taken. CellTracker Green-stained cells were manually counted as live cells and PI-stained cells as dead. Cell viability was calculated by correlating the number of live and dead cells with the total amount of cells.

### Cell Staining

At the end of each assay, the cells were fixed with 3.7% paraformaldehyde in PBS for 30 min at room temperature, followed by 4× washes with PBS. Cells were permeabilized with 0.1% Triton-X in PBS for 10 min and blocked with the first blocking solution (3% Bovine Serum Albumin and 0.3 M Glycine) for 30 min and the second blocking solution (5% goat serum in PBS) for 30 min. The samples were washed with PBS 3 times for 5 min with mild agitation in between each step. Cells were incubated with the mix of anti-YAP (sc-101199, Santa Cruz) and antivinculin (EPR8185, Abcam) primary antibodies overnight and secondary antibody mix for 1 h the following day. After cells were stained with phalloidin dye for actin staining for 15 min and DAPI dye for nuclear staining for 5 min. The samples were finally washed 4× with PBS, bonded with Mowiol solution to a coverslip, and left to dry for over 48 h. Imaging was performed by a high content screening microscope Nikon (Eclipse Ti2-D-PD).

### Analytical and Statistical Analysis

All reported cell data contain three independent experiments (*N* = 3). For cell imaging analysis for each condition, 20–25 images were acquired with 200–400 cells per condition. Cell morphology and antibody staining intensity were quantified by using Cell Profiler software. Data was plotted and analyzed by GraphPad Prism. Outlier analyses were performed on all data sets with the ROUT method, Q = 0.1%. One-way ANOVA with the nonparametric Kruskal–Wallis test was used to assess the differences between conditions with a significance level of 0.05.

## Supporting Information Available

Detailed information on chemicals and chemical synthesis used for the LC film fabrication; methods and parameters of characterization of the LC films; FTIR spectrum of the crude material (LC mix) versus the polymerized LC (LC film); DSC thermographs of the LC mix, LC topography formation in water at 37 °C; water contact angle of the LC film surface prior and post UV exposure; absorbance spectrum of an LC film at 37 °C in air indicating the decay of azobenzene; absorbance spectrum of the LC film before and after UV exposure compared to after visible light exposure; human dermal fibroblast cell morphology and phenotype on coverslip glass; quantification of the YAP and vinculin intensity and signal distribution on a coverslip glass; additional discussion of cell morphology and mechanosensing on flat LC films (PDF)
