# Supplementary material for: Light-Responsive Liquid Crystal Surface Topographies for Dynamic Stimulation of Cells
Source: ACS Appl Mater Interfaces. 2025 May 3;17(19):27871–81. doi: 10.1021/acsami.5c02526 (PMC12086769; doi:10.1021/acsami.5c02526)
Supplement: Supplementary file 1 — am5c02526_si_001.pdf [file am5c02526_si_001.pdf]

Supporting Information

Light-responsive liquid crystal surface topographies for dynamic stimulation of cells

Ruth M.C. Verbroekken[§,1,2], Oksana K. Savchak[§,2,3], Thom F.J. Alofs[1], Albert P.H.J. Schenning[*,1,2], Burcu Gumuscu[*,2,3]

[1]Stimuli-Responsive Functional Materials & Devices, Department of Chemical Engineering and Chemistry, Eindhoven University of Technology, PO Box 513, 5600 MB Eindhoven, The Netherlands.

[2] Institute for Complex Molecular Systems, Eindhoven University of Technology, P.O. Box 513, 5600 MB Eindhoven, The Netherlands.

[3]Biosensors and Devices Laboratory, Department of Biomedical Engineering, Eindhoven University of Technology, PO Box 513, 5600 MB Eindhoven, The Netherlands.

Corresponding authors: b.gumuscu@tue.nl and a.p.h.j.schenning@tue.nl

Experimental procedures

Chemicals: 2-methyl-1,4-phenylene bis(4((6-(acryloxy)hexyl)oxy)benzoate)) (**1**, RM82) was acquired from Daken chemical. 4-cyanophenyl 4-((6-(acryloyloxy)hexyl)oxy) benzoate (**2**, RM23) and 4-methoxyphenyl4-((6-(acryloyloxy)hexyl)oxy)benzoate (**3**, RM105) were purchased from Merck. 4,4'-bis(6-acryloyloxyhexyloxy)azobenzene (**4**, A6A) was purchased from SYNTHON Chemicals GmbH & Co. bis[4-[[4-[[[4-[(1-oxo-2-propenyl)oxy]butoxy]carbonyl]oxy]benzoyl]oxy]benzoate] (**5**, LC756) was obtained from BASF. 2,2'-(ethylenedioxy)diethanethiol 95% (**6**, DODT) was obtained from Sigma-Aldrich. Irgacure 819 (**7**, IRG819) was purchased from Ciba Specialty Chemicals NV. The solvent Dichloromethane (DCM, 99.9%) was obtained from Biosolve. For the functionalized glass plates, 3-(trimethoxysilyl)propyl methacrylate and 1H,1H,2H,2H-perfluorodecyltriethoxysilane were purchased from Sigma-Aldrich and ethanol from TechniSolv (100.0%). SP-220 20 μm spacer beads are acquired from Sekisui Chemical Co. LTD. UVS 91 UV-glue was acquired from Norland Products INC. All chemicals were used as received without further purification.

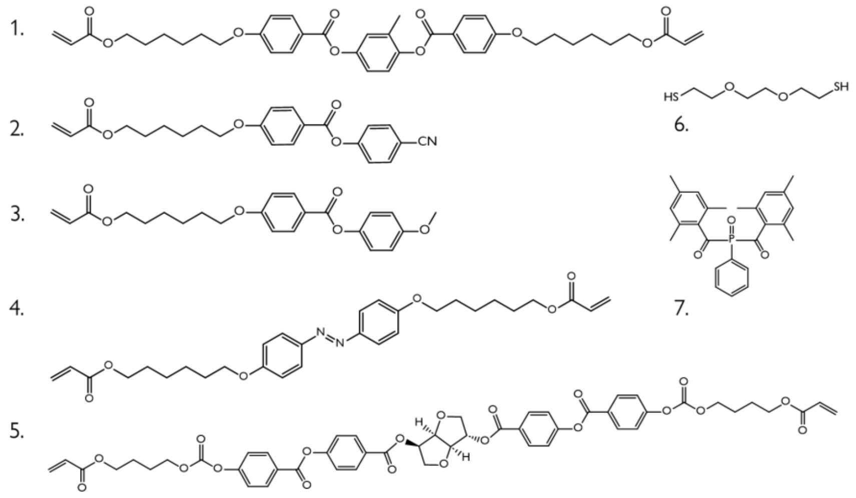

Figure S 1. *Compounds used for the preparation of the cholesteric LC film*

All reagents and chemicals were obtained from commercial sources and without further purification. For the fabrication of the LC films, a mixture (**Figure S1**) of A6A (8,0 wt%), LC756 (3,2 wt%), RM82 (5,0 wt%), RM23 (30,8 wt%), RM105 (45,6 wt%), Irgacure 819 (4,0 wt%), DODT (3,4 wt%) was dissolved in 2 mL dichloromethane (DCM). Overnight vacuuming of the LC mix removed the solvent after which yielding the final LC mix. Thin films of the light-responsive LC mix were prepared with a droplet LC mixt between two oxygen plasma treated and sequentially functionalized borosilicate glass

plates. The top plate was functionalized by spincoating a 2 vol% solution of 3-(trimethoxysilyl)propyl methacrylate in ethanol for LC film attachment to the surface. Whereas the bottom plate was functionalized by spincoating a 2 vol% solution of 1H,1H,2H,2H-perfluorodecyl-triethoxysilane in ethanol, ensuring easy removal of this plate post-polymerization. On the four corners between the glass slides, a UV curable glue with 20 µm spacer beads was applied, yielding ~20 µm thick films. Manual unidirectional shearing aligns the molecules in the film which is then fully photopolymerized for 15 minutes via 455 nm UV exposure with an intensity of 27 mW/cm^2 at 30°C, using a Thorlabs M455L3 mounted with a COP1-A collimator. Subsequently, the LC film was heated to 80 °C to post cure the material.

Characterization: The LC surface and present topographical features are characterized using the Sensofar S Neox white-light interferometer. For all measurements the 10x objective is used and a scan of 80 µm in the z-direction is performed with coherence scanning interferometry. The scans are made using the maximum sensitivity and a resolution of 5 MP. This results in a scan with a surface area of 1.69 x 1.41 mm. The surfaces are further analysed in the Sensoview 2.3.1 software. Here, some corrections can be made by adjusting the threshold or flattening the surface in the case that focus has shifted during the scan. 3D and 2D images are subsequently exported and by drawing a horizontal line over the topographies, a profile of these topographies is also extracted. For these measurements a peak-to-valley height of a single area of five pillars gives an insight into the topographical height decay.

Thermal properties were determined using a Q2000 Differential Scanning Calorimeter (DSC) produced by TA Instruments. A run with three heating and cooling cycles was used with a rate of 5 °C per minute. The LC mixture was measured between -50 °C and 100 °C and the LC film from -50 °C up to 150 °C. The analysis was done using the software from TA Instruments, Trios. For the analysis only the third and final cycle was considered to remove any thermal history in the sample.

The IR spectra of the LC mixture and film was obtained by a 670-IR spectrometer from Varian. The resulting spectrum is analyzed in SpectraGryph 1.2 where compared spectra are normalized to the carbonyl stretching peak, present in both the LC mixture and the polymerized LC film, at 1720-1740 cm^{-2}.

The transmission spectra of the samples in air are measured using a Perkin-Elmer lambda 750 UV/Vis/ NIR spectrophotometer. The spectra are measured between 250 and 1100 nm with a data interval of 1 nm. The glass plate is taken as the 100% transmittance background for the measurement. For the time-dependent measurements, the spectrometer was set to take a spectrum after a specified time for a set number of times. Measurements at 37°C were measured in combination with a Linkam heating stage and a T95 controller provided by Linkam Scientific instruments. UV-vis data

was processed in the Origin Software. Noisy data was smoothed using the smoothing function "Adjacent-Averaging" setting on 10.

Water contact angle measurements were conducted on a Dataphysics OCA30 goniometer, using MilliQ water and drops with a volume of 5 µL. Three measurements were gathered per condition and the contact angles comprise of the average between the individual left and right contact angles.

Material stiffness was measured using a Cypher Environmental Scanner atomic force microscope provided by Oxford instruments. The LC film stiffness in air at 37°C was measured in contact mode using a super luminescent diode to reduce the signal-to-noise ratio. A high-density diamond-like carbon probe is used, characterized by a spring constant of 2.8 N/m and a tip radius of 20 nm (Biosphere B20-FM – Nanotools). An area of 1 μm^2 was measured using the Fast Force Map mode with a scan rate of 0.31 Hz, a z-rate of 100.00 Hz and an oscillation amplitude of 400.00 nm.

The data was analysed and plotted using the software Origin 2022. Curve-fitting was done using the non-linear curve fit function in Origin with a custom formula.

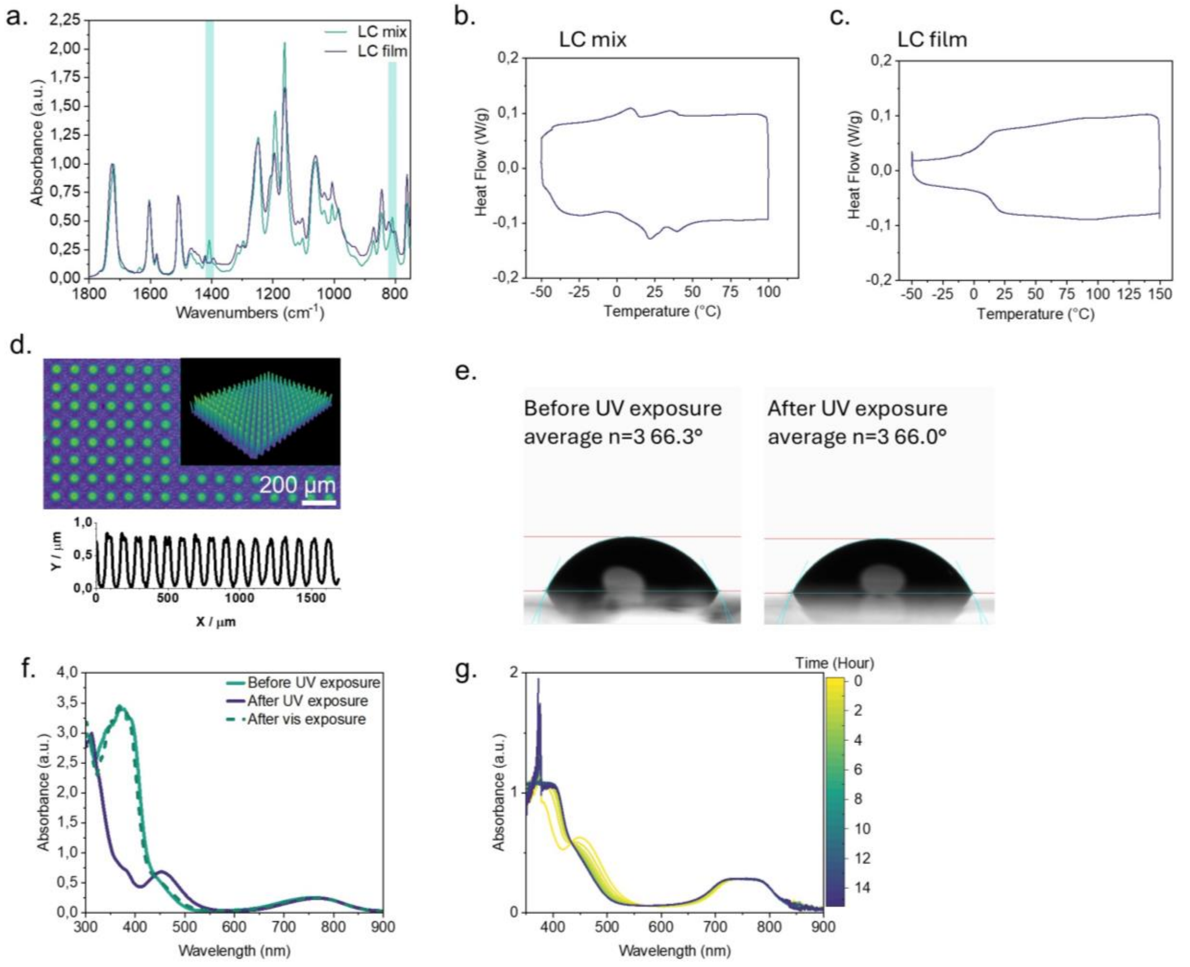

Figure S 2. a) *FTIR spectrum of the crude material (LC mix, green) versus the polymerized LC (LC film, purple). The marked regions indicate the absence of free-acrylate groups by the* $CH_2{=}CH_2$ *de-formation and* $CH{=}CH_2$ *twisting vibrations at 1410* cm^{-1} *and 810* cm^{-1}*, respectively. DSC thermo-graphs of the LC mix.* ***b)*** *Before polymerization as a crude material* ***c)*** *post polymerization, showing the third heating run.* ***d)*** *LC film dispersed in water at 37°C in air, showing topography formation of pillars (800 nm peak-to-valley height, 50 µm diameter features on 50 µm spacing).* ***e)*** *Water contact angle measurements before (left) and post (right) UV exposure, showing comparable water contact angles of ~66 °.* ***f)*** *Absorbance spectrum of the LC film before (green), after (purple) 4 min UV-illumination, and after subsequent 10 min visible light illumination (dashed-green) of an LC film.* ***g)*** *Absorbance spectrum of an LC film at 37°C in air indicating the cis-azobenzene decay post UV-actuation.*

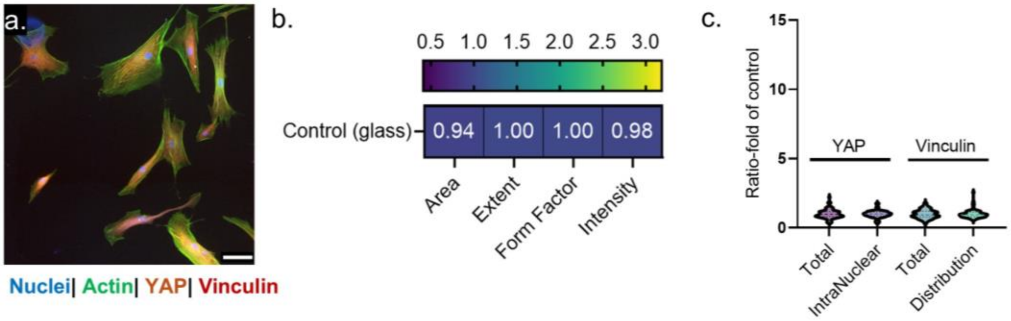

Figure S 3*. Human dermal fibroblast cell morphology and phenotype on coverslip glass.* ***a)*** *Fibroblast on coverslip glass fluorescent staining. The cell nuclei stained with blue, actin cytoskeleton with green, YAP protein with orange and vinculin focal adhesions with red. Scale bar is 100 µm.* ***b)*** *Heatmap of the morphology of the fibroblast cells on the coverslip glass. N = 3.* ***c)*** *Quantification of the YAP and vinculin intensity and signal distribution, N = 3. Y scale is matched to the Figure 4.*

Cell morphology and mechanosensing on flat LC films

Fibroblasts are known to adopt more compact and spherical shapes on softer surfaces as reported by Hackett et al., and Kessler et al.[1,2]. Since decrease in the cell size and increased circularity are observed on all LC films, we hypothesize that the morphological changes are majorly defined by the LC film stiffness parameter. Glass coverslips have been widely used for cell culture and have a reported range of stiffness between 20-60 GPa[3,4]. In contrast, the non-activated laminin-coated LC surface, measured by AFM, exhibited a stiffness of approximately 1.8 GPa, which decreased upon actuation to about 1.2 GPa—10 to 20 times softer than the glass coverslip surface. For example, the size and circularity of a cell is largely regulated by surface stiffness, where the stiffer surfaces promote more elongated cell shapes[5]. Additionally, surface uniformity and circularity can be indicative of the fibroblast' phenotype, where pro-reactive phenotypes tend to form more protrusions and adopt larger but less elongated shapes[6,7]. The approximate 0.5 GPa difference in stiffness between the non-actuated and fully actuated conditions raises the question of whether this variation can induce a differential cellular response. The stiffness of the LC film is lower than that of the glass coverslip surface; however, the difference between the non-actuated and actuated surfaces is unlikely to elicit a distinct cellular response. Natural biological tissues[8] and synthetic materials[3,5,9] used to study cell responses to stiffness changes typically exhibit stiffness in the kPa range. It has been observed that cell responses tend to plateau when stiffness exceeds the MPa range[10,11]. Therefore, the stiffness differences above MPa appear to not induce any notable difference in cell response. Due to this fact, the 0.5 GPa difference observed in between non-actuated and actuated LC film is considered negligible and is not expected to affect the cell response.

Due to the observed high level of mechanical stimulation, actin intensity, as well as a notable increase in focal adhesion expression and organization, we hypothesize that the LC films have spontaneous numerous small-scale actuations and decays due to the sample exposure to the ambient light. Cells can sense surface roughness as low as 10 nanometres,[12,13] and dynamic movement of the surface on even smaller temporal and spatial scales can trigger cell responses related to migration and re-modelling. In the reported results, the cells on the non-exposed control and fully exposed control experience numerous smaller events of surface modification that can activate the mechanical signal-ling as a response. Moreover, cells showing a strong mechanical response to flat LC film surfaces, challenge the paradigm of the smallest surface change that can be sensed by cells and opens a possibility of stretching the limit of sensitivity of cells to a mechanical stimulus.

References

(1) Hackett, T. L.; Vriesde, N. R. T. F.; Al-Fouadi, M.; Mostaco-Guidolin, L.; Maftoun, D.; Hsieh, A.; Coxson, N.; Usman, K.; Sin, D. D.; Booth, S.; Osei, E. T. The Role of the Dynamic Lung Extracellular Matrix Environment on Fibroblast Morphology and Inflammation. *Cells* 2022, *11* (2). https://doi.org/10.3390/cells11020185.

(2) Kessler, D.; Dethlefsen, S.; Haase, I.; Plomann, M.; Hirche, F.; Krieg, T.; Eckes, B. Fibroblasts in Mechanically Stressed Collagen Lattices Assume a “Synthetic” Phenotype. *Journal of Biological Chemistry* 2001, *276* (39), 36575–36585. https://doi.org/10.1074/jbc.M101602200.

(3) Acevedo-Acevedo, S.; Crone, W. C. Substrate Stiffness Effect and Chromosome Missegregation in HIPS Cells. *J Negat Results Biomed* 2015, *14* (1). https://doi.org/10.1186/s12952-015-0042-8.

(4) Seal, A.; Dalui, A. K.; Banerjee, M.; Mukhopadhyay, A. K.; Phani, K. K. Mechanical Properties of Very Thin Cover Slip Glass Disk. *Bulletin of Materials Science* 2001, *24* (2), 151–155. https://doi.org/10.1007/BF02710092.

(5) Solon, J.; Levental, I.; Sengupta, K.; Georges, P. C.; Janmey, P. A. Fibroblast Adaptation and Stiffness Matching to Soft Elastic Substrates. *Biophys J* 2007, *93* (12), 4453–4461. https://doi.org/10.1529/biophysj.106.101386.

(6) Li, B.; Wang, J. H. C. Fibroblasts and Myofibroblasts in Wound Healing: Force Generation and Measurement. *J Tissue Viability* 2011, *20* (4), 108–120. https://doi.org/10.1016/j.jtv.2009.11.004.

(7) Darby, I. A.; Laverdet, B.; Bonté, F.; Desmoulière, A. Fibroblasts and Myofibroblasts in Wound Healing. *Clin Cosmet Investig Dermatol* 2014, *7*, 301–311. https://doi.org/10.2147/CCID.S50046.

(8) Wei, Q.; Wang, S.; Han, F.; Wang, H.; Zhang, W.; Yu, Q.; Liu, C.; Ding, L.; Wang, J.; Yu, L.; Zhu, C.; Li, B. Cellular Modulation by the Mechanical Cues from Biomaterials for Tissue Engineering. *Biomaterials Translational* 2021, *2* (4), 323–342. https://doi.org/10.12336/biomatertransl.2021.04.001.

(9) Isomursu, A.; Park, K. Y.; Hou, J.; Cheng, B.; Mathieu, M.; Shamsan, G. A.; Fuller, B.; Kasim, J.; Mahmoodi, M. M.; Lu, T. J.; Genin, G. M.; Xu, F.; Lin, M.; Distefano, M. D.; Ivaska, J.; Odde, D. J. Directed Cell Migration towards Softer Environments. *Nat Mater* 2022, *21* (9), 1081–1090. https://doi.org/10.1038/s41563-022-01294-2.

(10) Khounsaraki, G. M.; Movahedi, M.; Oscuii, H. N.; Voloshin, A. Analysis of the Adherent Cell Response to the Substrate Stiffness Using Tensegrity. *Ann Biomed Eng* 2024, *52* (5), 1213–1221. https://doi.org/10.1007/s10439-024-03447-7.

(11) Chiang, M. Y. M.; Yangben, Y.; Lin, N. J.; Zhong, J. L.; Yang, L. Relationships among Cell Morphology, Intrinsic Cell Stiffness and Cell-Substrate Interactions. *Biomaterials* 2013, *34* (38), 9754–9762. https://doi.org/10.1016/j.biomaterials.2013.09.014.

(12) Loesberg, W. A.; te Riet, J.; van Delft, F. C. M. J. M.; Schön, P.; Figdor, C. G.; Speller, S.; van Loon, J. J. W. A.; Walboomers, X. F.; Jansen, J. A. The Threshold at Which Substrate Nanogroove Dimensions May Influence Fibroblast Alignment and Adhesion. *Biomaterials* 2007, *28* (27), 3944–3951. https://doi.org/10.1016/j.biomaterials.2007.05.030.

(13) Dalby, M. J.; Riehle, M. O.; Johnstone, H.; Affrossman, S.; Curtis, A. S. G. Investigating the Limits of Filopodial Sensing: A Brief Report Using SEM to Image the Interaction between 10 Nm High Nano-Topography and Fibroblast Filopodia. *Cell Biol Int* 2004, *28* (3), 229–236. https://doi.org/10.1016/j.cellbi.2003.12.004.
